# Supplementary material for: Inbreeding depression causes reduced fecundity in Golden Retrievers
Source: Mamm Genome. 2019 May 21;30(5):166–72. doi: 10.1007/s00335-019-09805-4 (PMC6606663; doi:10.1007/s00335-019-09805-4)
Supplement: Supplementary file 1 — Supplementary material 1 (DOCX 286 kb) [file 335_2019_9805_MOESM1_ESM.docx]

# **SUPPLEMENTARY MATERIAL**

**Inbreeding depression causes reduced fecundity in Golden Retrievers.** Erin T. Chu[1, 3, ORCID [0000-0003-1193-7387](https://orcid.org/0000-0003-1193-7387)], Missy J. Simpson [4, ORCID [0000-0002-9411-8039](http://orcid.org/0000-0002-9411-8039)], Kelly Diehl [4], Rodney L. Page [4,5, ORCID [0000-0002-6094-7541](https://orcid.org/0000-0002-6094-7541)], Aaron J. Sams*[1, 3, ORCID [0000-0002-3800-4459](http://orcid.org/0000-0002-3800-4459)], and Adam R. Boyko*[1,2,3]

[1] Embark Veterinary, Inc., 184 Lincoln St, 6th Floor, Boston, MA 02111
[2] Department of Clinical Sciences, Cornell University College of Veterinary Medicine, Ithaca, NY 14853
[3] Corresponding authors, chue@embarkvet.com, +1 (607) 229-2306, asams@embarkvet.com, adam@embarkvet.com
[4] Morris Animal Foundation, Denver, CO 80246
[5] Flint Animal Cancer Center, Colorado State University, Fort Collins, CO 80523

# **SUPPLEMENTARY FIGURES**

**
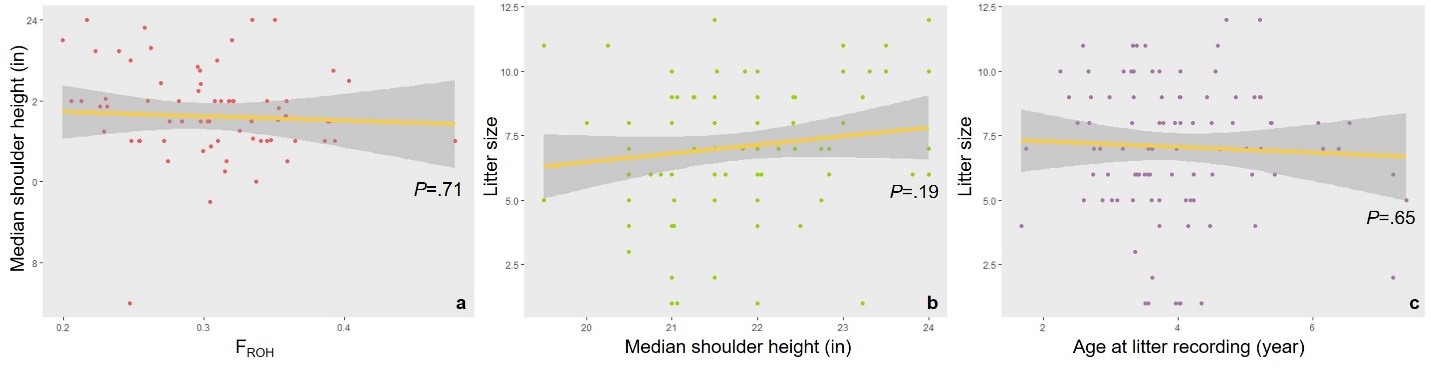
**

**Fig S1**. **Linear regression plots of a)** median shoulder height against F_ROH_, **b)** litter size against median height, and **c)** litter size against age at time of litter recording. While correlations between all three sets of factors have been demonstrated in the literature, we do not observe significant correlations between them in this dataset.

**
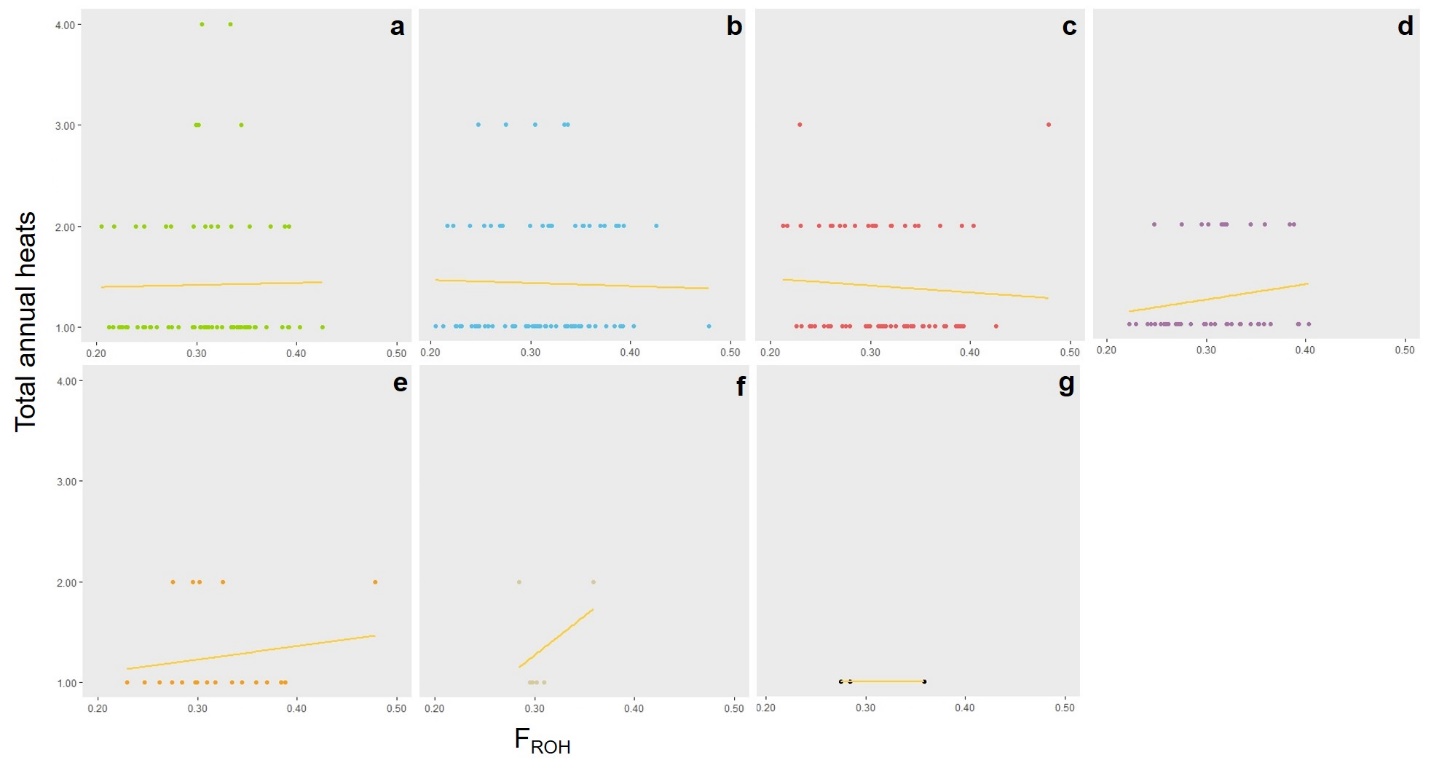
**

**Fig S2.** **Regression of estrous cycles (heats) recorded against F_ROH_ for Embark-GRLS dogs at a) 1, b) 2, c) 3, d) 4, e) 5, f) 6, and g) 7 years of age**. Linear regressions are included in yellow on each plot. Recorded heats range from 1 to 4 heats per year at any age with no significant correlation between with F_ROH_ and annual heats at any year of age. Data points decrease dramatically for 6 (tan) and 7 (black) year old dogs.

**
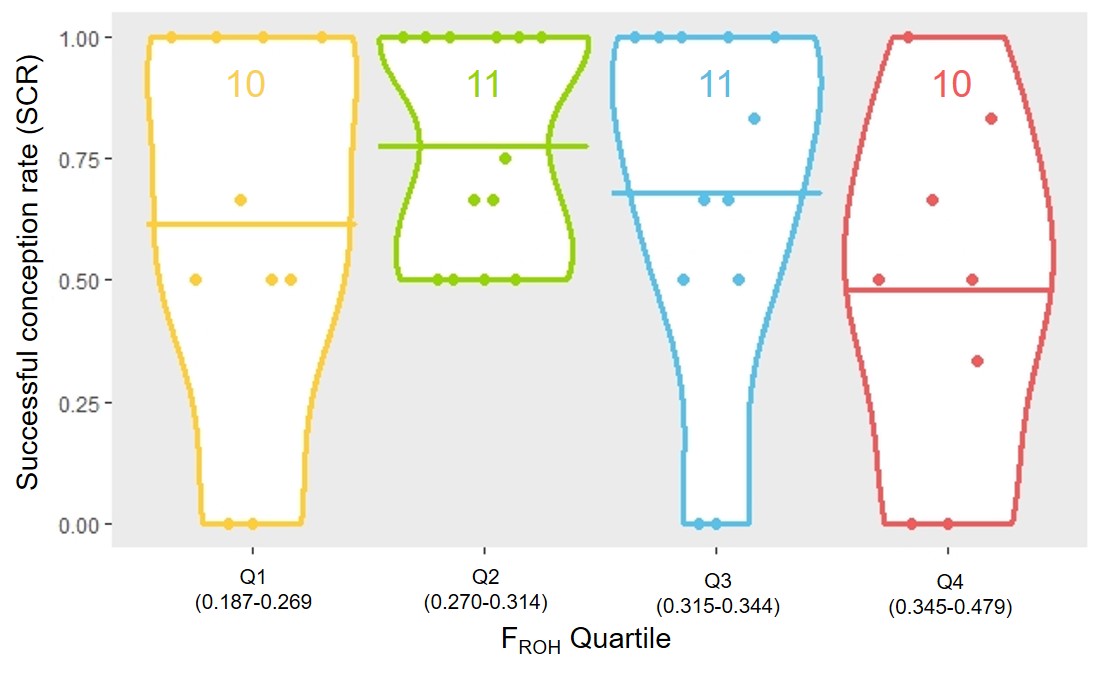
**

**Fig S3.** **Violin plots of SCR of GRLS dogs who had been bred at least twice (n=44) separated into F_ROH_ quartiles**. Number of observations per quartile are indicated in each violin plot. Crossbars represent average SCR within quartiles. Dogs in the fourth quartile (0.344 < F_ROH_ < 0.479) have a lower average SCR than dogs in the first, second, and third quartiles, though this difference is insignificant (*P*=.21).


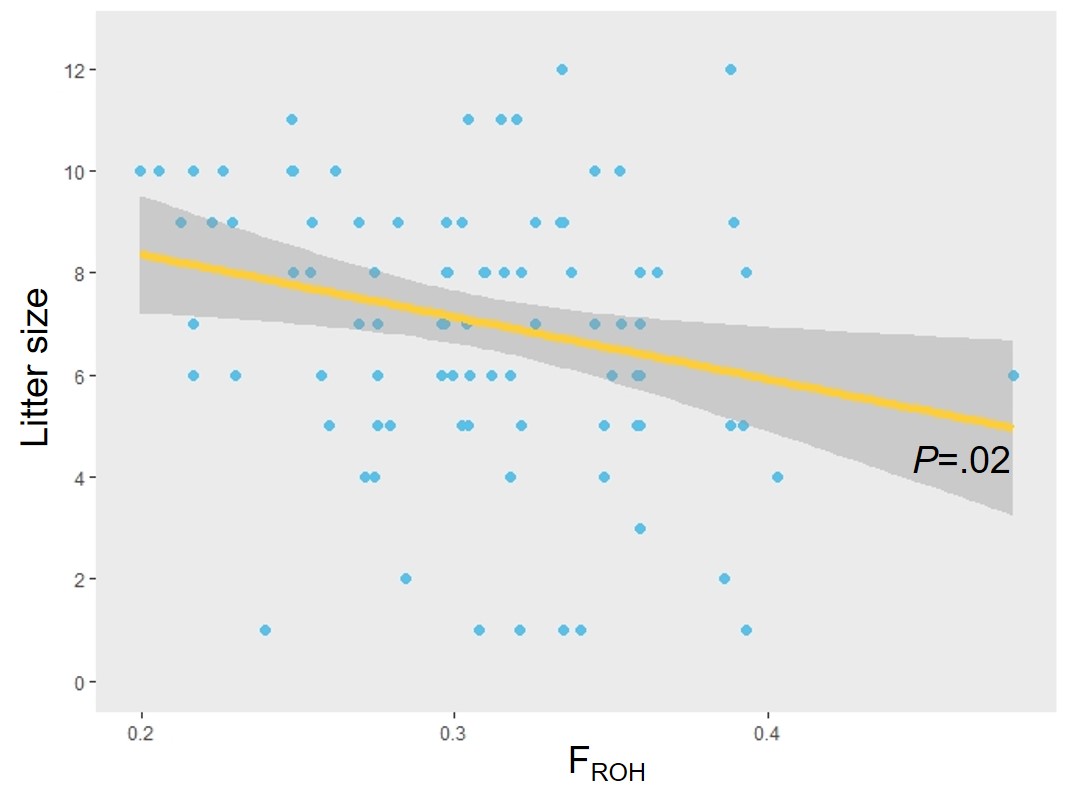


**Fig S4.** Linear regression of litter size as measured by live puppies as a function of F_ROH_. Litter size is inversely correlated with F_ROH_. Individual litters are plotted in blue; linear regression (R^2^=0.102, P=.02) is shown in yellow with 95% confidence interval in gray.

# **SUPPLEMENTARY TABLES**

**Table S1.** Summarizing table of statistics on the 100 dogs selected for the Embark-GRLS cohort. Note that not all dogs represented in this table were genotyped; their statistics are not included in this study.

|  | **Integer Age (years)** | | | | | | |
| --- | --- | --- | --- | --- | --- | --- | --- |
| **Characteristic** | **1** | **2** | **3** | **4** | **5** | **6** | **7** |
| Number of visits | 63 | 133 | 136 | 109 | 46 | 19 | 6 |
| Median body condition score (min, max) | 4(4,7) | 4(2,7) | 5(4,7) | 5(2,6) | 5(2,8) | 5(3,7) | 5(5,7) |
| Mean Height at shoulders in cm (±sd) | 54.7(3.2) | 55.2(3.2) | 54.9(3.1) | 54.6(2.6) | 54.9(2.3) | 53.8(1.5) | 54.6(2.0) |
| Mean Weight in kg (±sd) | 25.4(3.8) | 26.7(3.5) | 27.9(3.4) | 28.3(3.2) | 27.6(3.7) | 28.8(4.5) | 28.9(4.8) |
| Total breedings | 1 | 13 | 58 | 62 | 27 | 8 | 3 |
| Total litters | 0 | 7 | 41 | 42 | 20 | 5 | 3 |
| % of breeding resulting in litter | 0% | 54% | 71% | 68% | 74% | 63% | 100% |
| Total live births (min, max) | n/a | 59(4,11) | 273(2,11) | 251(2,10) | 143(2,12) | 32(7,10) | 13(2,6) |
| Total stillborn (min, max) | n/a | 3(0,3) | 24(0,3) | 33(0,5) | 7(0,2) | 1(0,1) | 2(0,2) |
| Total weaned (min. max) | n/a | 56(4,10) | 227(0,10) | 241(1,10) | 125(2,12) | 32(7,10) | 13(2,6) |
| Number of c-sections (% of pregnancies) | n/a | 2(28%) | 13 (32%) | 9(21%) | 4(20%) | 0 | 0 |
